# Isoniazid-induced polyneuropathy in a tuberculosis patient – implication for individual risk stratification with genotyping?

**DOI:** 10.1002/brb3.326

**Published:** 2015-05-29

**Authors:** Mark Stettner, Daniela Steinberger, Christian J Hartmann, Tatjana Pabst, Lidija Konta, Hans Peter Hartung, Bernd C Kieseier

**Affiliations:** 1 Department of Neurology, Medical Faculty, Heinrich-Heine-UniversityDüsseldorf, Germany; 2 bio.logis Center for Human Genetics, Frankfurter Innovationszentrum Biotechnologie (FIZ)Frankfurt am Main, Germany; 3 Institute of Human Genetics, Justus-Liebig-UniversityGiessen, Germany

**Keywords:** c1/c2, CYP2E1, individual risk stratification, isoniazid, linezolid, N-acetyltransferase 2, *NAT2*, polyneuropathy, side effects, slow acetylators, toxic effects, tuberculosis

## Abstract

**Background:**

Development of polyneuropathy (PNP) under treatment for tuberculosis (TB), including isoniazid (INH), is a highly relevant adverse drug effect. The *NAT2* acetylation status is a predictor of potential toxic effects of INH. The question as to whether individual risk stratification by genotyping is useful to avoid suffering of patients and to lower costs for the health care system is of considerable clinical importance.

**Case Presentation:**

After drug treatment for TB, including INH, a 23-year-old man developed severe PNP. During the treatment, laboratory results have been indicating incipient liver and renal injury. Later, molecular genetic analyses were performed and revealed a variation in the *NAT2* gene and the *c1/c2* genotype of the CYP2E1 gene, both described to contribute to an elevated risk for anti-tuberculostatic-induced liver damages (ATIL).

**Conclusion:**

The combination of metabolizer genotypes should be taken into account as a cause for toxic effects and the development of PNP. Individual genotyping, performed before medication or at least if an elevation of liver parameters is observed, may reduce the risk of severe cases of PNP by early adjustment of treatment. Our case study indicates that evaluation of individual risk stratification with systematic pharmacogenetic genotyping of metabolizer gene combinations in the context of TB treatment should be addressed in clinical studies with larger cohorts.

## Background

Development of PNP under treatment for tuberculosis (TB) is a frequent and significant adverse drug effect; a putative cause for inducing PNP is isoniazid (INH). However, ethambutol may induce PNP in very rare cases with regression after medication has been suspended (Donomae and Yamamoto 1966; Tugwell and James 1972), streptomycin more frequently causes ototoxicity than PNP (Argov and Mastaglia 1979). After prolonged treatment, linezolid is known to contribute to PNP development, particularly in combination with other PNP-inducing drugs. Recent reports state that the injury may be irreversible (Ntziora and Falagas 2007).

The incidence of PNP in INH-treated patients in cross-sectional studies revealed a rate ranging from 2 to 44% for an HIV-negative group (Breen et al. 2006; Marks et al. 2009). This distinction is relevant due to the fact that new cases of TB mainly occur in Asia and Africa in association with HIV, which regularly induces PNP either by itself or via HART, for example, treatment with nucleoside-reverse transcriptase inhibitors (NRTI) (Schifitto et al. 2002; Breen et al. 2006; Marks et al. 2009; Maritz et al. 2010).

The pathophysiological background of INH-induced PNP is not well understood. It is likely that isonicotinic acid hydrazide interferes with vitamin B6 (pyridoxine) metabolism, leading to deficiency in biological active B6 by inhibition of pyridoxine-dependent enzyme systems (Biehl and Vilter 1954; Snider 1980; Preziosi 2007). This fact necessitates pyridoxine supplementation under treatment with INH. Various studies have reported that an increased blood concentration of INH resulted in an increased risk of adverse effects, including PNP (Krishnamurthy et al. 1967; Matar et al. 2004; Preziosi 2007; Wang et al. 2012). The acetylator status has a direct impact on the blood concentration of the drug, and consequently on the efficacy as well as the toxic effects of the drug. Thus, the potential for the development of PNP under INH is higher for slow inactivators (Krishnamurthy et al. 1967). For metabolization and inactivation of INH, N-acetyltransferase type 2 (*NAT2*) is crucial (Evans et al. 1960). INH plasma concentrations are higher (up to six-fold at short intervals) in those individuals with low-activity alleles of *NAT2* (Parkin et al. 1997; Kinzig-Schippers et al. 2005).

Furthermore, a significant association between *NAT2* acetylation status and the risk of developing antituberculosis drug-induced liver injury (ATLI) was observed (Wang et al. 2012). However, the relevance of molecular genotyping of the NAT2 gene and the development of adverse effects is critically discussed in the literature. A study in a Mexican population found an association between the acetylator genotype and phenotype, but no association was found between the genotype or phenotype and the incidence of INH-related adverse reactions (Azuma et al. 2013).

The frequencies of slow and fast acetylators are equal in most European Caucasian populations (van der Watt et al. 2011). The combined risk with other genes associated with INH metabolism has not been elucidated so far.

## Case Presentation

A 23-year-old man was referred as an outpatient to our clinic 21 months after diagnosis of TB with neurological symptoms encompassing severe pain, paraesthesia and hypoesthesia following TB treatment.The patient’s history revealed that he developed severe productive coughing with yellow expectoration and haemoptysis after his return from a backpacking trip in Asia. The X-ray of his chest revealed infiltration of the superior part of the right lung including a cavernous structure. The sputum was positive for acid-fast rods, further diagnostics including HIV-testing resulted in normal findings.

The patient was initially treated with ethambutol, INH (with pyridoxine supplementation), rifampicin, pyraniazid and streptomycin. He developed elevated liver enzymes (gamma glutamyltransferase (GGT) 119 U/L, aspartate aminotransferase (AST) 100 U/L, alanine aminotransferase (ALT) 314 U/L) as well as elevation of the retention parameters (creatinine 1.4 mg/dL). After 1 month, resistance testing revealed a multidrug-resistant infection; the treatment was amended to capreomycin, linezolid, and moxifloxacin, and treatment with ethambutol was continued. Two months after the initial diagnosis, the patient complained of paraesthesia of his feet.

During the following year, the symptoms worsened and the patient was referred to a regional hospital for further PNP diagnostics. Neither analysis of the cerebrospinal fluid (CSF indicated a total protein of 26 mg/dL, lactate 1.7 mmol/L, glucose 62 mg/dL, and no pleocytosis) nor serologic PNP screening revealed a pathological result. Since the patient’s condition worsened, symptomatic treatment with pregabalin was initiated, but symptoms hardly decreased.

**Figure 1:**
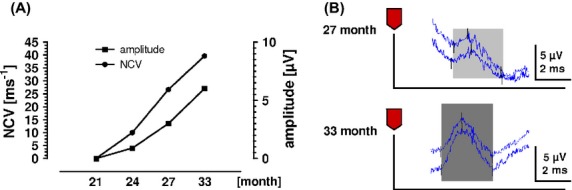
(A) Nerve conduction velocity study of the sural nerve, time points indicated in month after TB diagnosis. A period of 21 months after diagnosis of TB the patient was examined for the first time, nerve conduction studies improved in follow-up neurography and revealed normal results 33 months after the initial diagnosis. (B) Exemplary graphs of nerve conduction velocity study 27 an 33 months after TB diagnosis.

One and a half years after diagnosis of TB, the patient was referred to our hospital; TB treatment was already successfully completed. Nerve conduction studies of the sural nerve revealed amplitudes that were severely reduced or absent, a pattern that improved in follow-up neurography over a time period of 2 years (Fig.1). Four years after the initial diagnosis of TB, the patient was free of neurological symptoms.

In order to explore the potential cause of the individual toxic effect of TB drugs, molecular genetic analyses of genes that are known to harbor variations relevant to drug effects were analyzed. Genotyping of *ABCB1*, *CYP1A2*, *CYP3A4*, *CYP2A6*, *CYP2C9*, *SLCO1B1,* and *UGT1A1* revealed unremarkable results concerning a causative effect for adverse drug reactions.

A polymorphism c.590G>A was detected in heterozygous state in the *NAT2* gene, resulting in the genotype NAT2*4/*6. This genotype is known to be associated with reduced activity (intermediate acetylator, IA) of the N-acetyltransferase 2 enzyme encoded by the gene. The enzyme is important for the metabolism and inactivation of active drug substances such as INH into inactive metabolites. Reduced enzyme activity leads to increased concentrations of INH and a higher risk of toxic effects such as injury of internal organs and PNP (Singla et al. 2004).

For the *CYP2E1* gene, the *c1/c2* genotype was detected and the*2-allele as well as the *1D-allele excluded. It was recently reported that the *c1/c2* genotype contributes toward an elevated risk of tuberculosis drug-induced toxicity (Singla et al. 2004). In the East Asian population, the *c1/c1* genotype (high activity) is associated with drug-induced liver damage. About 80% of Asians present the *c1/c1* genotype.

## Conclusion

Our patient developed signs of incipient organ injury indicated by elevated laboratory parameters for liver and kidney. Under consideration of the INH-treatment, these have to be interpreted as the first signs of overdose and toxic levels of the active substance, which finally culminate in the neurological condition.

The elevation of liver enzyme levels did not fulfil the criteria for drug-induced hepatotoxicity, defined as the serum level of aspirate aminotransferase >3 times the upper limit of the normal with symptoms (or five times the upper limit of normal without symptoms) (Blumberg et al. 2003), and so the therapy was not modified. However, this paraclinical sign was the first evidence of an elevated INH blood concentration, which can be regarded as a precondition of the PNP. It is noteworthy that there are still no reliable data defining the impact of individual metabolic enzyme levels and pyridoxine requirements under INH, and hence the risk of developing PNP.

In the current case, we cannot completely rule out a role of linezolid in the development of the patient’s PNP, and it may have exacerbated the INH-induced nerve damage. The patient did not receive linezolid during the increase in enzyme levels, and the latency of the appearance of clinical symptoms suggests that INH was the primary agent.

There is consensus that TB-treatment with INH results in a certain risk of developing PNP. It has already been suggested that molecular screening of patients for variants of *NAT2* might be useful for the clinical prediction and prevention of ATLI (Wang et al. 2012).

Another pharmacogenetic clinical trial found that an NAT2 genotype-guided adjustment of INH treatment led to lower incidences of adverse events such as isoniazid-related liver injury or early treatment failure than a conventional standard regimen. It is surprising that there was no statistically significant difference in the incidence of polyneuropathy between the NAT2 genotype-guided treatment and the standard treatment group (Diaz-Molina et al. 2008). This result, and the fact that in some studies no association was found between the genotype or phenotype and the incidence of INH-related adverse reactions (Azuma et al. 2013), may be due to neglect of the observation that the combination of genotypes is a key factor in identifying patients with greater susceptibility to the development of such adverse reactions.

Genotyping for combinations of common genetic variants associated with drug metabolism before medication or at least after the observation of initial indicators for potentially toxic levels of INH such as abnormal conventional laboratory parameters under standard dosage may reduce the individual risk of severe cases of PNP. Since the costs of genetic analyses are decreasing in a remarkable dynamic and variant-specific amendment of therapy leads to lower costs for the health care system, reducing the suffering of patients with these techniques can be justified on economic as well as ethical reasons.

For *NAT2*, recent studies also suggest a significant percentage of intermediate acetylators*,* resulting in adverse effects under anti-tuberculosis treatment (Singla et al. 2004). The intermediate acetylator haplotype for *NAT2* in combination with other genotypes that contribute to increased plasma levels of the drug (such as *c1/c1* for CYP2E1) suggest an even greater risk.

Further clinical studies are necessary to evaluate the effect of combined genotypes for individual pharmacogenetic risk stratification on a regular basis.

## Consent

Written informed consent was obtained from the patient for publication of this case report and any associated images. A copy of the written consent is available for review from the Editor of this journal.

## Conflict of Interest

None declared.
